# Standardized local assortativity in networks and systemic risk in financial markets

**DOI:** 10.1371/journal.pone.0292327

**Published:** 2023-10-05

**Authors:** Mike K. P. So, Anson S. W. Mak, Jacky N. L. Chan, Amanda M. Y. Chu

**Affiliations:** 1 Department of Information Systems, Business Statistics and Operations Management, The Hong Kong University of Science and Technology, Hong Kong, Hong Kong; 2 Faculty of Science, University of Amsterdam, Amsterdam, The Netherlands; 3 Department of Social Sciences and Policy Studies, The Education University of Hong Kong, Hong Kong, Hong Kong; Lodz University of Technology: Politechnika Lodzka, POLAND

## Abstract

The study of assortativity allows us to understand the heterogeneity of networks and the implication of network resilience. While a global measure has been predominantly used to characterize this network feature, there has been little research to suggest a local coefficient to account for the presence of local (dis)assortative patterns in diversely mixed networks. We build on existing literature and extend the concept of assortativity with the proposal of a standardized scale-independent local coefficient to observe the assortative characteristics of each entity in networks that would otherwise be smoothed out with a global measure. This coefficient provides a lens through which the granular level of details can be observed, as well as capturing possible pattern (dis)formation in dynamic networks. We demonstrate how the standardized local assortative coefficient discovers the presence of (dis)assortative hubs in static networks on a granular level, and how it tracks systemic risk in dynamic financial networks.

## Introduction

Network analysis is a common technique used to detect and understand relationships among entities [1], and is employed in a variety of fields such as physics [2–4], social sciences [5, 6], biological, medical and epidemiological studies [7–13], business [14], and finance [15–19]. A network graph is made up of nodes that depict entities of a system and edges that indicate pairwise relationships. A graph is weighted if the edges between nodes have different strengths or attributes.

There are various measures for quantifying network structures, such as network degree and clustering coefficients to summarize the network size and the embeddedness of nodes in networks, respectively. Assortativity, or assortative mixing [4], is another measure used to quantify networks and tells us how likely nodes are to connect with other nodes that are similar to themselves. Sociological studies often describe assortative mixing as *homophily*, a phenomenon often found in a variety of social networks, among humans [20–22] and in nature [23]. In homophilous behavior, people tend to bond with others who are similar to themselves when they share common socio-demographic, behavioral, and intra-personal characteristics, such as gender, ethnicity, hobbies, and values, resulting in homogeneous personal networks for each individual [21]. Human networks are dynamic and individuals tend to form bonds with cooperators and break ties with defectors [22]. Constant formations and dissolutions of relationships form a continuously evolving human ecology. The evolution and ubiquity of homophily is affected by natural selection in that through collaborations and interactions with similar others, individuals create synergy and provide complementary skills to increase potential gains [24]. Over time, humans have learned to cooperate with assortative others and thereby achieve higher payoffs [22, 25].

Assortativity has implications for network resilience, in terms of how robust and reliable a network remains in the event of faults and operational challenges with some of its nodes [26]. In most networks, the connectivity can be destroyed by simply removing a few nodes with the highest number of edges. Consider technology hubs or service providers—they are usually the first targets of attacks because the failure of a few important “hubs” will create a domino effect that can bring down all services connected to them. Therefore, the study of network resilience is of high importance in the field of computer science, especially for highly critical systems in today’s digital age [26, 27].

Technological networks, along with biological and neural networks, are considered disassortative, suggesting that they are especially vulnerable and likely to be prone to targeted attacks [4]. Conversely, the assortative nature of social networks allows them to be more resilient, and their structure is therefore not easily disrupted. In the case of transmittable diseases, core groups in assortatively mixed social networks can form a “reservoir” for diseases that could sustain a pandemic [4]. As a result, pandemics have been found to be robust in face of simple targeted attacks on social networks, such as isolating a few infected individuals who have high degrees of connections in the midst of a pandemic [28]. Other studies have looked at regional networks to reveal the recent example of how the coronavirus disease 2019 (COVID-19) pandemic spread rapidly across the globe given the network’s connectedness [29, 30].

Global assortativity (GA) [3, 4, 31–33] measures the assortative mixing of networks and provides us with a glimpse of several aspects of those networks. In particular, the research provides significant insights into the relationship between global assortativity and network robustness, as well as the role of global assortativity in the evolution of cooperation in networks [31–33]. However, the coefficient is only representative if most of the nodes of a network have a degree of assortative mixing around the mean, and in some networks that is not the case [34]. When networks grow, thanks to technological advancement and easier data collection and storage, it is not hard to imagine that these complex networks could have more diverse local (dis)assortative mixing. As a measurement of averages, local assortative patterns are smoothed out and therefore might not present a full picture of the relationships in the networks.

A number of studies have proposed the use of local assortativity [34, 35] to overcome the shortcomings of a global measure, but there has not been a universal definition of how such a local coefficient should be calculated. With the lack of a local coefficient, it is difficult to compare and trace local assortativity features that might tell us more about the networks and reveal previously undiscovered patterns. In response, this study provides examples of when the global assortativity coefficient fails to address networks with local assortative hubs. We further extend the concept of local assortativity based on the existing literature, as well as proposing a standardized formula that we believe is an important add-on because it allows for comparisons on the same scale. We show in the *Discussion* section how this standardized local assortativity coefficient helps to explain the networks and the potential predictive power it provides.

This paper makes the following contributions. First, we adopt a local assortativity measure from the literature and provide a standardized version of it in light of the limitations that this measure might have when the network size grows. We then compare the standardized local coefficient against the global one in several networks of different fields to highlight its significance. Last, we investigate how a standardized local assortativity could provide predictive power in a dynamic network setting, and shed light on tracking systemic risk in financial markets.

## Materials and methods

### Assortative mixing

The commonly used approach to measuring the assortative mixing of a network is the framework proposed by Newman [3], which measures whether nodes tend to attach to each other based on the similarity in their degrees. We define *p*_*ij*_ as the joint probability distribution of the degrees between nodes *v*_*i*_ and *v*_*j*_ that satisfies the following sum rules:

$$\begin{array}{c} \sum_{ij}p_{ij}=1\;\sum_{j}p_{ij}=a_{i}\;\sum_{i}p_{ij}=b_{j} \end{array}$$ (1)

where *a*_*i*_ and *b*_*j*_ represent the fraction of edges that start and end at nodes *v*_*i*_ and *v*_*j*_, respectively. If no assortative mixing is found in the network, *p*_*ij*_ = *a*_*i*_*b*_*j*_. Otherwise, the global assortativity, *GA*, is defined as:

$$\begin{array}{c} GA=\frac{\sum_{ij}ij\left(p_{ij}-a_{i}b_{j}\right)}{\sigma_{a}\sigma_{b}} \end{array}$$ (2)

where *σ*_*a*_ and *σ*_*b*_ denote the standard deviations of *a*_*i*_ and *b*_*j*_, respectively [4]. *GA* has a range of −1 ≤ *GA* ≤ 1, with 1 indicating perfect assortativity and -1 is perfect disassortativity. An estimate of *GA* in Eq (2) can be found in [3].

*GA* describes the assortativity of a network on a global level and is essentially the Pearson’s correlation coefficient of the degree between nodes. However, in networks that show a mix of assortative and dis-assortative patterns, *GA* might not give an accurate picture. To look at local assortativity, *LA*_*j*_ of a random node *v*_*j*_, we adopt the formula in [35]:

$$\begin{array}{c} LA_{j}=\frac{1}{d_{j}}\sum_{i=1}^{d_{j}}\left|d_{j}-d_{j(i)}\right| \end{array}$$ (3)

where *d*_*j*_ is the degree of node *v*_*j*_, nodes *v*_*j*(*i*)_ for *i* = 1, …, *d*_*j*_ are the neighbour nodes linking to node *v*_*j*_, and *d*_*j*(*i*)_ is the degree of the node *v*_*j*(*i*)_.

This calculation is computationally less exhaustive, and assumes that all nodes in a network are, to a certain degree, disassortative. As local assortativity is a relative concept, the authors [35] argue that the average number of differences in node degrees between any node *j* and its neighbors can therefore be seen as the disassortativity, that is, how disassortative node *j* is when compared with its neighbouring nodes. Therefore, local assortativity, or *LA*_*j*_, can be expressed by Eq (3).

A shortcoming of *LA*_*j*_ is that it is not scale-invariant, thus making the comparison of *LA*_*j*_ among nodes dependent upon the values of *d*_*j*_. Consider a simple example having nodes *j* and *j*′ that *d*_*j*′_ = *cd*_*j*_, *d*_*j*′(*i*)_ = *cd*_*j*(*i*)_ and |*d*_*j*_ − *d*_*j*(*i*)_| = *M*, where *c* and *M* are positive constants. It is obvious that nodes *j* and *j*′ have the same assortative properties, but $LA_{j^{\prime }}=\frac{1}{d_{j^{\prime }}}\sum_{i=1}^{d_{j^{\prime }}}\left|d_{j^{\prime }}-d_{j^{\prime }\left(i\right)}\right|=cM=cLA_{j}$. In addition, if nodes have a huge difference in their degree, which is not uncommon in the case of complex networks, *LA*_*j*_ can be heavily skewed given its unbounded range of [0, +∞). In view of this, we propose a standardized version of *LA*_*j*_, *SLA*_*j*_ to account for the issue. Given $|d_{j}-d_{j(i)}{|}^{2}\leq d_{j}^{2}+d_{j(i)}^{2}$, we standardized the coefficient by dividing the summation of node degree differences in *LA*_*j*_ by $\sum_{i=1}^{d_{j}}\sqrt{d_{j}^{2}+d_{j(i)}^{2}}$,

$$\begin{array}{c} SLA_{j}=\frac{\sum_{i=1}^{d_{j}}\left|d_{j}-d_{j(i)}\right|}{\sum_{i=1}^{d_{j}}\sqrt{d_{j}^{2}+d_{j(i)}^{2}}}. \end{array}$$ (4)

Because of the normalization by the denominator $\sum_{i=1}^{d_{j}}\sqrt{d_{j}^{2}+d_{j(i)}^{2}}$ instead of the denominator *d*_*j*_ in *LA*_*j*_, *SLA*_*j*_ is scale-invariant.

**Fig 1:**
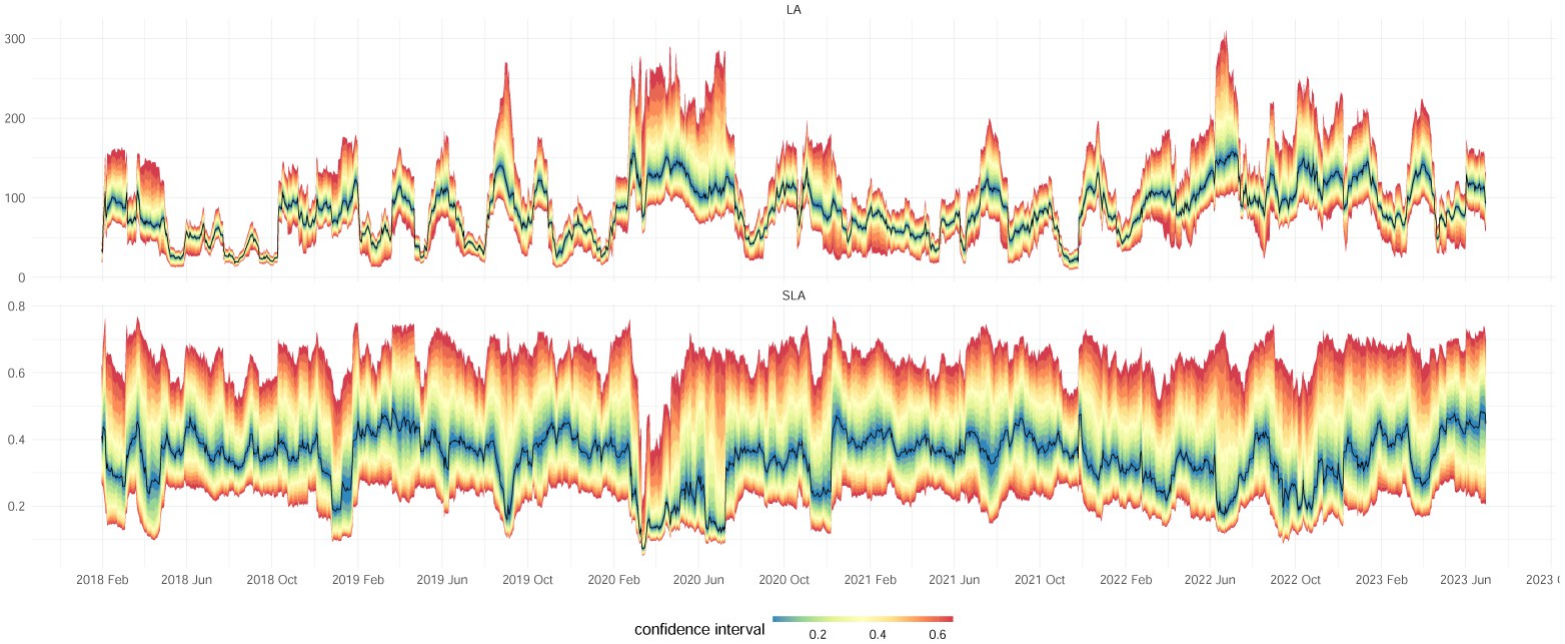
The effects of standardizing the local assortativity, *LA*_*j*_ in Eq (3) to *SLA*_*j*_ in Eq (4), and their confidence intervals. The results were obtained by constructing a dynamic financial network using daily financial returns from global markets.

Fig 1 shows the effect of standardizing the coefficient in a dynamic financial network constructed using financial returns data. More details of the financial network construction are described in the section on “Data Collection”. The coefficient *LA*_*j*_ is an unbounded coefficient, whose value is scale-dependent on the difference in degrees between node *j* and its neighboring nodes. Values of *SLA*_*j*_, on the other hand, lies within the range of 0 ≤ *SLA*_*j*_ ≤ 1 as $|d_{j}-d_{j(i)}{|}^{2}\leq d_{j}^{2}+d_{j(i)}^{2}$. This provides a neater summary description of the network. According to the construction in Eq (4), when *SLA*_*j*_ approaches one, node *v*_*j*_ will be highly disassortative. When *SLA*_*j*_ approaches zero, node *v*_*j*_ will be highly assortative. To facilitate the comparison with the global assortativity, *GA*, we define the standardized local assortativity of node *v*_*j*_ as

$$\begin{array}{c} Q_{j}^{\left(s\right)}=1-2SLA_{j}, \end{array}$$ (5)

making $-1\leq Q_{j}^{\left(s\right)}\leq 1$, with larger/smaller $Q_{j}^{\left(s\right)}$ implying that node *v*_*j*_ is more locally assortative/disassortative. In an extreme case where *d*_*j*_ > 0 and all *d*_*j*(*i*)_ are zeros implying that node *j* is highly dissortative, $Q_{j}^{\left(s\right)}=-1$. In another extreme case where *d*_*j*_ > 0 and *d*_*j*_ = *d*_*j*(*i*)_ implying that node *j* is highly assortative, $Q_{j}^{\left(s\right)}=1$. In this paper, we will continue with this defined standardized local assortativity coefficient, $Q_{j}^{\left(s\right)}$, to investigate network structures in the multiple examples, including the one shown in Fig 1.

### Data collection

To demonstrate the significance of the standardized local assortativity coefficient, we made use of three networks from different fields of studies: (1) the Lancichinetti-Fortunato-Radicchi benchmark (LFR benchmark), (2) a Facebook ego, (3) a scale-free network, and (4) dynamic financial networks. The LFR benchmark network has 1000 nodes and was generated with the parameters shown in Table 1. We obtained the open Facebook ego network data from the Stanford Network Analysis Project, and it contains survey data from anonymized Facebook participants [36]. Each node represents an individual with their own profile attributes, and edges are the pairwise affiliations between individuals. To construct the dynamic financial networks, we used two data sources from Reuters. We collected the daily adjusted closing price of the constituents of five selected worldwide stock market indices, using the Reuters Terminal API: 1) Hong Kong’s Hang Seng Index (HSI50), 2) the United Kingdom’s Financial Times Stock Exchange 100 Index (FTSE100), 3) the United States’ Standard and Poor’s 500 (SP500), 4) France’s CAC40, and 5) Germany’s DAX30, from January 2018 to June 2023. Because the constituents of the indices might change over time, we manually collected a list of all historical constituents of the indices from the Reuters terminal. Only a few records could not be mapped due to unlisted stock information, and we were able to use the remaining 1158 constituents at a different time *t* for further analysis in our study.

**Table 1:** Parameter setting for LFR benchmark network.

Parameter	Value
Number of nodes	1000
Exponent for degree distribution	3
Exponent for community size distribution	1.1
Fraction of inter-community edges incidence	0.06
Minimum degree of nodes	10
Maximum degree of nodes	50
Minimum size of communities	10
Maximum size of communities	50

### Construction of financial networks

Using the daily closing price *P*_*j*,*t*_ of stock *j* on trading day *t*, we calculated the return of stock *j* at time *t* by

$$\begin{array}{c} Y_{jt}=log\left(P_{j,t}\right)-log\left(P_{j,t-1}\right). \end{array}$$ (6)

We defined dynamic undirected networks *G*_*t*_ = (*V*_*t*_, *E*_*t*_) [30], where *V*_*t*_ is the set of nodes and *E*_*t*_ is the set of edges at time *t*. We calculated the sample Pearson correlation between stocks *i* and *j* at time *t*, *ρ*_*ij*,*t*_, using a method often referred to as a “rolling-window” approach [37], and collecting the past 21 days of historical observations (*Y*_*i*,*t*−*k*_, *Y*_*j*,*t*−*k*_) for *k* = 0, …, 20. To construct a financial network at time *t*, an edge *e*_*ij*,*t*_ between two nodes *v*_*i*_ and *v*_*j*_ is created if *ρ*_*ij*,*t*_ between stocks *i* and *j* at time *t* is greater than 0.5 [30].

$$\begin{array}{c} e_{ij,t}=\{\begin{array}{cc} 1 & \;\text{if}\;\rho_{ij,t}>0.5,\\ 0 & \text{otherwise}. \end{array} \end{array}$$ (7)

The rolling-window approach enables the use of the most recent information to represent the network information at time *t*.

### Network statistics

We made use of the degree of node *v*_*j*_ at time *t*, *d*_*j*,*t*_, to investigate whether it could complement the local assortativity coefficient to predict future losses in dynamic financial networks. The degree *d*_*j*,*t*_ describes the number of connections in the network *G*_*t*_,

$$\begin{array}{c} d_{j,t}=\sum_{i\in V_{t}}e_{ij,t}. \end{array}$$ (8)

The network density in Table 3 was calculated as

$$\begin{array}{c} \frac{\sum_{j=1}^{V_{t}}d_{j,t}}{V_{t}\left(V_{t}-1\right)}, \end{array}$$ (9)

which is defined as the total number of edges in the network *G*_*t*_ divided by the total number of possible edges with *V*_*t*_ nodes. We also followed [3, 30] to calculate the global assortativity coefficients listed in Table 3, as in Eq (10).

$$\begin{array}{c} \frac{V_{t}^{-1}\sum_{i=1}^{V_{t}}\sum_{j=1}^{V_{t}}d_{i,t}d_{j,t}e_{ij,t}-{\left[V_{t}^{-1}\sum_{i=1}^{V_{t}}\sum_{j=1}^{V_{t}}\frac{1}{2}\left(d_{i,t}+d_{j,t}\right)e_{ij,t}\right]}^{2}}{V_{t}^{-1}\sum_{i=1}^{V_{t}}\sum_{j=1}^{V_{t}}\frac{1}{2}\left(d_{i,t}^{2}+d_{j,t}^{2}\right)e_{ij,t}-{\left[V_{t}^{-1}\sum_{i=1}^{V_{t}}\sum_{j=1}^{V_{t}}\frac{1}{2}\left(d_{i,t}+d_{j,t}\right)e_{ij,t}\right]}^{2}}. \end{array}$$ (10)

The global assortativity coefficient in Eq (10) can be interpreted as the degree correlation. It is calculated by using all pairs of degrees whose nodes are linked together to calculate the correlation coefficient. Therefore, it describes how strong degrees of linked nodes are related linearly with each other in the whole network (globally), rather than describing the local assortativity feature by $Q_{j}^{\left(s\right)}$. In other words, the global assortativity coefficient is dominated by nodes with high degrees. In addition, if nodes that are highly assortative/disassortative have high degrees, the global assortativity tends to be positive/negative. On the other hand, the scale-invariant $Q_{j}^{\left(s\right)}$ measure will not be affected by the scale of the node degrees, thus making the distribution of $Q_{j}^{\left(s\right)}$ more objective in determining the assortativity of networks than the global assortativity coefficient is.

### Correlation between standardized local assortativity and future volatility

With the daily closing price *P*_*j*,*t*_ of stock *j* at time *t*, we calculated the *k*-period return *k* at time *t*,

$$\begin{array}{c} Y_{j,t+k}=log\left(P_{j,t+k}\right)-log\left(P_{j,t}\right) \end{array}$$ (11)

where *k* = 1, 2, 3, …. We correlated $Q_{j,t}^{\left(s\right)}$ with the absolute *k*-period return, |*Y*_*j*,*t*+*k*_| of stock *j* at time *t*, with *k* = 1, …, 10 to investigate the predictability of $Q_{j,t}^{\left(s\right)}$ for future volatility using |*Y*_*j*,*t*+*k*_| as a proxy:

$$\begin{array}{c} q_{jk,t}=Corr(Q_{j,t}^{\left(s\right)},|Y_{j,t+k}\left|\right). \end{array}$$ (12)

For each combination of *j*, *k* and *t*, we estimated *q*_*jk*,*t*_ using the sample correlation based on the paired data of the past 21 days, that is, $\left(Q_{j,t-h}^{\left(s\right)},|Y_{j,t-h+k}|\right)$, *h* = 0, …, 20, where *h* denotes a time lag within the past 21 days. Then, for each *k*, we produced the distribution of the correlation in Fig 5 using estimated *q*_*jk*,*t*_.

### Multiple correlations between standardized local assortativity and future losses

To investigate the predictability of future losses using the standardized local assortativity, we defined the loss of stock *j* at time *t* + 1 as

$$\begin{array}{c} L_{j,t+1}=\{\begin{array}{cc} -Y_{j,t+1} & \;\text{if}\;Y_{j,t+1}<0,\\ 0 & \;\text{otherwise}. \end{array} \end{array}$$ (13)

With that, we obtained the multiple correlation between $Q_{j,t-l}^{\left(s\right)}$, *l* = 0, ⋯, *N*, and the loss *L*_*j*,*t*+1_ by

$$\begin{array}{c} \begin{array}{cc} r_{y,x} & =\sqrt{\frac{S_{yx}S_{xx}^{-1}S_{xy}}{s_{y}^{2}}}\\ & =\sqrt{R_{yx}R_{xx}^{-1}R_{xy}}, \end{array} \end{array}$$ (14)

where *y* denotes *L*_*j*,*t*+1_, ***x*** denotes the predictor variables $Q_{j,t-l}^{\left(s\right)}$ for *l* = 0, ⋯, *N*, depending on the number of lags *N* to include, ***S***_*xy*_ denotes the sample covariance matrix between ***x*** and *y*, ***S***_*yx*_ denotes the transpose of ***S***_*xy*_, ***S***_*xx*_ denotes the sample covariance matrix between the predictor variables, $S_{xx}^{-1}$ denotes the inverse of ***S***_*xx*_, $s_{y}^{2}$ denotes the sample variance of *y*, ***R***_*xy*_ denotes the sample correlation matrix between ***x*** and *y*, ***R***_*yx*_ is the transpose of ***R***_*xy*_, ***R***_*xx*_ denotes the sample correlation matrix of the predictor variables, and $R_{xx}^{-1}$ denotes the inverse of ***R***_*xx*_. For each stock *j* and time *t*, the sample covariance and correlation matrices were calculated using data from the past 21 days, that is, $\left(Q_{j,t-h-n}^{\left(s\right)},n=0,\cdots ,N\right)$ and |*L*_*j*,*t*−*h*+1_|, for *h* = 0, ⋯, 20. We present the distribution of the multiple correlation for *N* = 0, ⋯, 5 in Fig 6.

With the additional inclusion of network statistics, *d*_*j*,*t*_, we calculated the multiple correlations between *L*_*j*,*t*+1_ and $Q_{j,t-l}^{\left(s\right)}$, *d*_*j*,*t*−*l*_, *l* = 0, ⋯, *N*, using Eq (14), except ***x*** here denotes variables $Q_{j,t-l}^{\left(s\right)}$ and *d*_*j*,*t*−*l*_ for *l* = 0, ⋯, *N*.

### Conditional multiple correlations between standardized local assortativity and future losses given $Q_{j,t}^{\left(s\right)}>\gamma$

The conditional multiple correlations between $Q_{j,t-l}^{\left(s\right)}$, *l* = 0, ⋯, *N*, and the loss *L*_*j*,*t*+1_ given $Q_{j,t}^{\left(s\right)}>\gamma$ were also calculated using Eq (14) but ignoring those days *t* in which the condition $Q_{j,t}^{\left(s\right)}>\gamma$ was not satisfied. In this paper, we consider *γ* = 0.6 as the threshold of high standardized local assortativity.

## Results

### Static networks

To compare the global and local assortativity coefficients, we made use of three static networks in this study: the Lancichinetti-Fortunato-Radicchi (LFR) benchmark, a Facebook ego network, and a scale-free network.

#### Lancichinetti-Fortunato-Radicchi (LFR) benchmark

**Fig 2:**
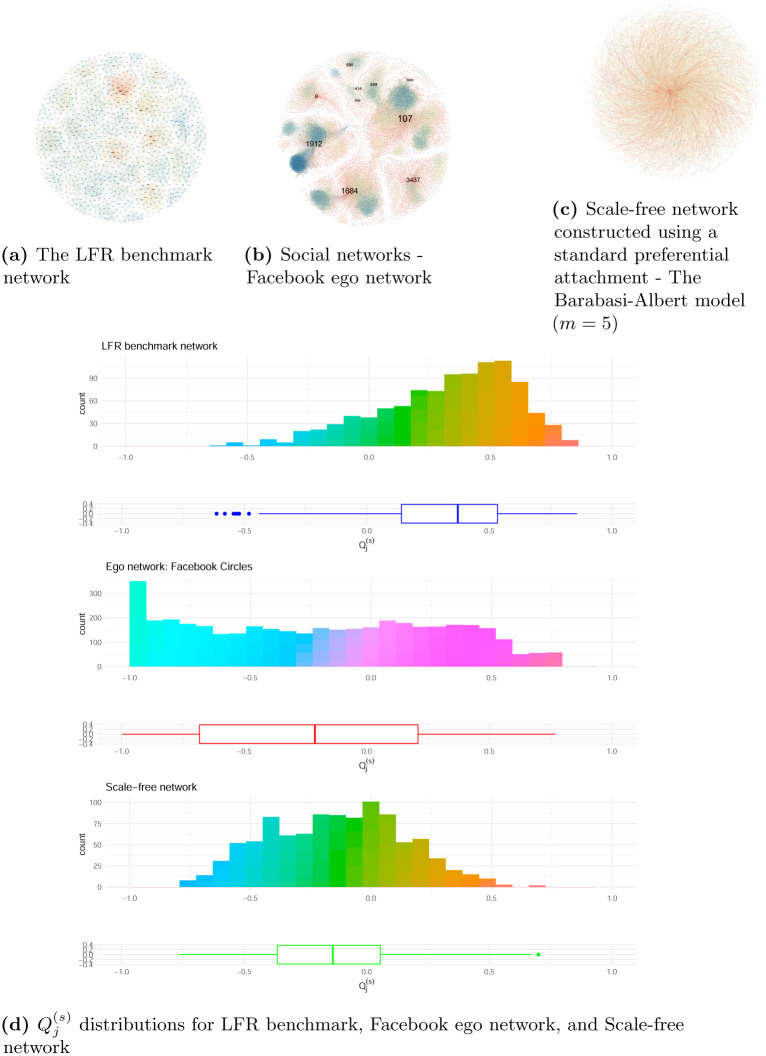
Three static networks used in this study and their $Q_{j}^{\left(s\right)}$ distributions. (a) The LFR benchmark. (b) Social networks—Facebook ego network. (c) Scale-free network. (d) The $Q_{j}^{\left(s\right)}$ distribution for the LFR benchmark, Facebook ego network and Scale-free network. The color of each node in the network plots in (a) through (c) represents the value of $Q_{j}^{\left(s\right)}$, ranging from -1 to 1 on a gradient scale: red represents -1 (highly disassortative), yellow represents 0, and blue represents 1 (highly assortative).

The LFR benchmark is a computer-generated network with prior known communities for comparing community detection methods. It is considered a proxy of real networks as it is capable of generating heterogeneous distributions of node degrees that follow the power law and the community size [38]. As such, generated networks will have assortative groups, depending on parameter settings. Fig 2a shows an example of a generated LFR benchmark network with 1000 nodes and 4850 edges. Summarized in Table 2, this network had an assortativity coefficient of −0.0002, suggesting that the benchmark network was neither assortative nor disassortative, despite the visible observation of closely related clusters in the network at a high level.

**Table 2:** Global assortativity *GA* and the median and mean standardized local assortativity $Q_{j}^{\left(s\right)}$ for the three static networks.

Network	GA	Median $Q_{j}^{\left(s\right)}$	Mean $Q_{j}^{\left(s\right)}$
LFR Benchmark	−0.0002	0.3718	0.3184
Facebook Ego Network	0.0636	−0.2110	−0.2163
Scale-free Network	−0.0022	−0.1382	−0.1424

#### Facebook ego network

The second network we considered was a network of social circles, or friend lists, from Facebook. It contains anonymized data from surveyed users [39]. We chose ego networks for our research because they are especially known for being assortative in some common social attributes, such as gender, age, and political views. Respondents, or *egos*, are asked to list the people that are directly linked to them, or *alters*, and to further describe the social relationships between them based on a variety of attributes. The extracted data set contained 10 combined ego networks, each of which if looked at separately showed a fairly strong assortative pattern, with *GA* between 0.084 and 0.503. This aligns with what the current literature suggests that social networks tend to be assortative in nature [21]. The combined network, which contained 4039 nodes and 88234 edges, as shown in Fig 2b, had a global assortativity of 0.0636 (Table 2), despite it containing multiple assortative sub-groups of individuals. This drastic change indicates that the global measure *GA* might not be an adequate coefficient for identifying local patterns in enormous networks.

#### Scale-free network

We constructed a scale-free network using a standard preferential attachment (the Barab*á*si-Albert model) to examine its assortativity properties. We set the number of edges that were added from a new node to existing nodes in each step of the network’s growth to be 5. Fig 2c shows the scale-free network with the global assortativity in Table 2 of -0.0022, which is close to zero. Here, therefore, the global measure *GA* shows no obvious assortative or disassortative trend globally.

#### Application of $Q_{j}^{\left(s\right)}$ on the three networks

With the limitations of *GA* in mind, we calculated $Q_{j}^{\left(s\right)}$ for each node *v*_*j*_ for the LFR benchmark, the Facebook ego network, and the scale-free networks. The second column of Table 2 shows the median values of $Q_{j}^{\left(s\right)}$ for the three networks. The median $Q_{j}^{\left(s\right)}$ values were 0.3718, -0.2110 and -0.1382 for the LFR benchmark, the Facebook ego network, and the scale-free network, respectively, while the Facebook ego network showed slightly more non-assortative mixing among its nodes. If we look at the $Q_{j}^{\left(s\right)}$ distributions in Fig 2d, we can observe that the LFR benchmark network is comparatively more skewed to the left, with more nodes showing local assortative patterns, and the proportion of positive values of $Q_{j}^{\left(s\right)}$ was 85%. On the other hand, the $Q_{j}^{\left(s\right)}$ distribution of the Facebook ego network revealed a small portion of nodes with an assortative pattern, such that the proportion of positive $Q_{j}^{\left(s\right)}$ was approximately 38%. Other nodes appear to have fairly uniform distributions except for the nodes that had a $Q_{j}^{\left(s\right)}$ value of nearly −1. With $Q_{j}^{\left(s\right)}=-1$ representing high disassortativity, the nodes included in the leftmost bar in the $Q_{j}^{\left(s\right)}$ distribution of the Facebook ego networks were highly unlikely to connect with other nodes with similar degrees. These nodes could potentially be the *egos* and mutual friends that acted as “hubs” linking diverse groups of friends in the network. In the scale-free network, the distribution of $Q_{j}^{\left(s\right)}$ is more symmetrical than it was in the LFR benchmark and Facebook ego networks. The median $Q_{j}^{\left(s\right)}$ was -0.1382, suggesting a locally disassortative mixing pattern. In other words, there was small tendency for nodes to connect to others with a different degree from them though *GA* is close to zero. Table 2 also presents the mean values of $Q_{j}^{\left(s\right)}$ which are consistent with what we observe from the median $Q_{j}^{\left(s\right)}$.

### Dynamic financial networks

The same limitation of a global coefficient is believed to apply to financial networks. One interesting feature of financial networks being added to the picture is that these networks are dynamic, that is, changes in the stock prices and indices occur within days, if not hours [17, 19]. The highly varying nature of the dynamic in financial markets leads to constant changes to the network structures and it is more challenging, but still important, to capture them.

**Fig 3:**
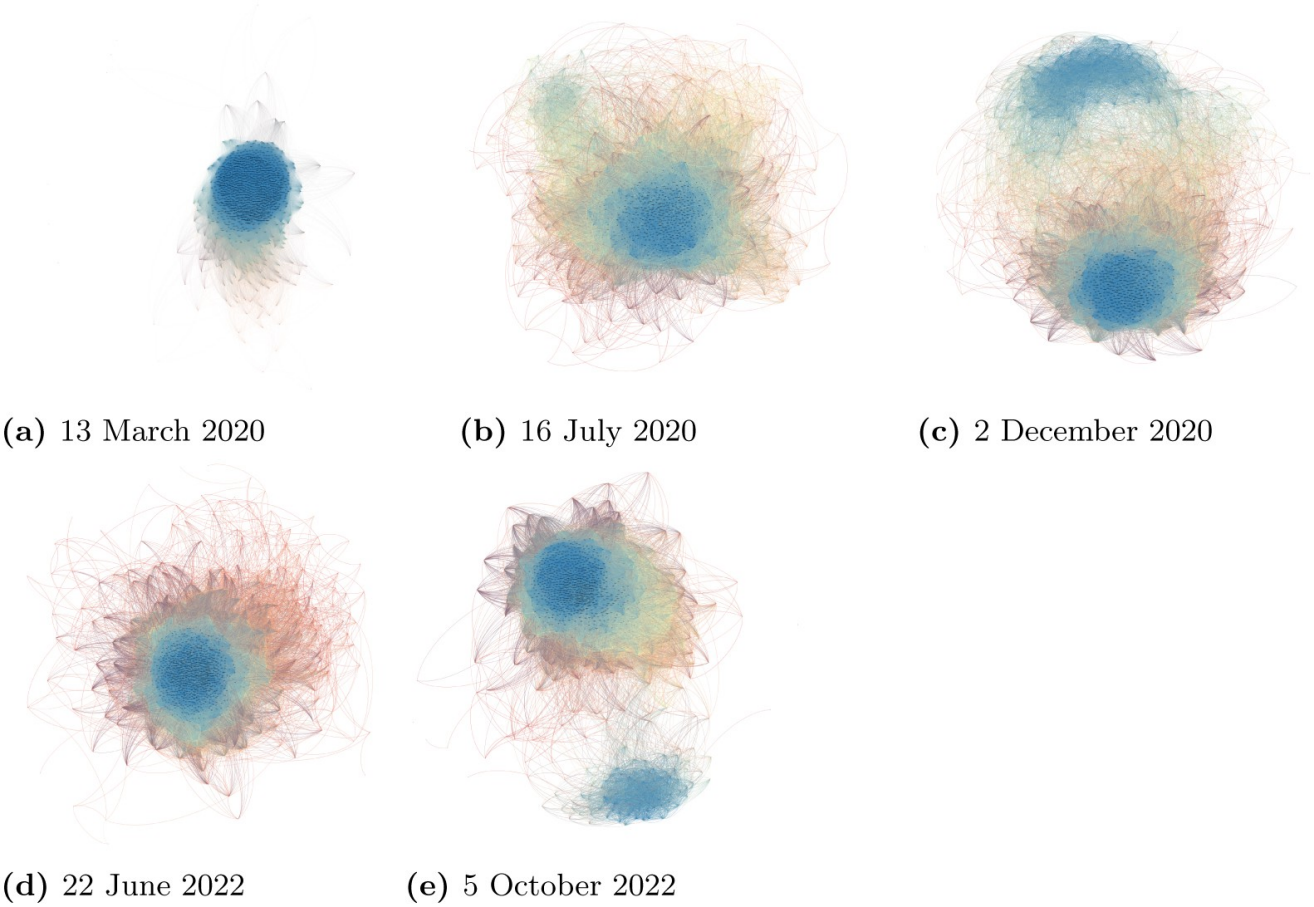
An illustration of financial networks on five randomly selected trading days. (a) 13 March 2020. (b) 16 July 2020. (c) 2 December 2020. (d) 22 June 2022. (e) 5 October 2022; The color of each node in the networks represents the value of $Q_{j}^{\left(s\right)}$, ranging from -1 to 1 on a gradient scale: red represents -1 (highly disassortative), yellow represents 0, and blue represents 1 (highly assortative).

**Fig 4:**
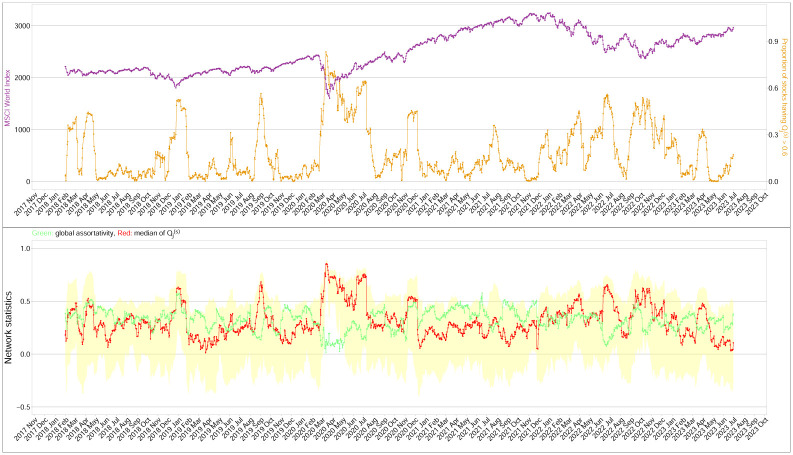
Top panel: the time series plot of the MSCI World Index from January 2018 to June 2023 (showing the worldwide financial market performance), and the proportion of stocks having $Q_{j,t}^{\left(s\right)}$ greater than 0.6 at each time point; Bottom panel: plots of the global assortativity (green line) and the median $Q_{j,t}^{\left(s\right)}$ (red line) with its interquartile ranges highlighted by yellow regions.

As an illustration, Fig 3 shows networks on several different trading days *t*. Table 3 presents the network density, global assortativity and the median $Q_{j,t}^{\left(s\right)}$ of the financial networks at the five selected time points. The standardized local assortativity $Q_{j,t}^{\left(s\right)}$ was obtained from the dynamic financial networks, as detailed in the *Materials and Methods* section. In Fig 3a, the network on 13 March 2020 exhibited an unusually high network density with the lowest global assortativity among the selected dates. The financial network became less dense as time went by, and eventually separated into two big clusters at the end of 2020, as shown in Fig 3c. The network at that time had the lowest network density, but the highest global assortative mixing compared with the other networks. The first selected date of 13 March 2020 was two days after the World Health Organization (WHO) had declared COVID-19 to be a global pandemic. On that day, with the financial market greatly affected by the COVID-19 pandemic, the global assortativity (0.14) was very different from the median standardized local assortativity, $Q_{j,t}^{\left(s\right)}$ (0.86). This demonstrates that our standardized local assortativity contains supplementary information on network assortative mixing. On the other two selected dates in 2020 when the financial market was relatively stable, the global assortativity and the median standardized local assortativity are relatively more consistent with each other. Table 3 also shows the network statistics for two selected dates in 2022, when we again note a large discrepancy between the global assortativity and the median $Q_{j,t}^{\left(s\right)}$. Possibly due to the tensions in Ukraine, the worry of economic recession and interest rate increases, the global financial markets dropped to their lowest points in the year around these two dates. In the three dates when the *GA* and the median $Q_{j,t}^{\left(s\right)}$ were very different, the mean $Q_{j,t}^{\left(s\right)}$ has value smaller than the corresponding median because the distribution of $Q_{j,t}^{\left(s\right)}$ was skewed to the left. Fig 3 and Table 3 thus demonstrate that when the financial market is very volatile due to an unexpected shock, global assortativity and $Q_{j,t}^{\left(s\right)}$ can be largely inconsistent, and the global assortativity may indicate spuriously low assortative mixing among stocks. Fig 4 presents the MSCI World Index, the proportion of $Q_{j,t}^{\left(s\right)}>0.6$ at each time point, the time series of the global assortativity, and the median and interquartile range of $Q_{j,t}^{\left(s\right)}$. There was severe downside movement in the MSCI in January, December 2018, March 2020 and June, October 2022, triggering potentially high systemic risk. In the five downside movements, the median and lower and upper quartiles of $Q_{j,t}^{\left(s\right)}$ increased sharply. The proportion of $Q_{j,t}^{\left(s\right)}>0.6$ also increased quickly when there was an extreme market adjustment. Even more striking observations were in March 2020 during the outbreak of COVID-19, when we note that the median and both quartiles of $Q_{j,t}^{\left(s\right)}$ were above 0.8. There were also obvious peaks in the proportion of $Q_{j,t}^{\left(s\right)}>0.6$ in June and October 2022. Together, these findings motivate us to anticipate that $Q_{j,t}^{\left(s\right)}$ could provide useful information for tracing systemic risk in financial markets, and thus to consider the empirical study we discuss next.

**Table 3:** Network statistics of five randomly selected dates.

Trading date, *t*	Network density	GA	Median $Q_{j,t}^{\left(s\right)}$	Mean $Q_{j,t}^{\left(s\right)}$
2020-03-13	0.73	0.14	0.86	0.67
2020-07-16	0.27	0.26	0.35	0.20
2020-12-02	0.23	0.48	0.51	0.28
2022-06-22	0.46	0.10	0.66	0.33
2022-10-05	0.41	0.25	0.62	0.41

**Fig 5:**
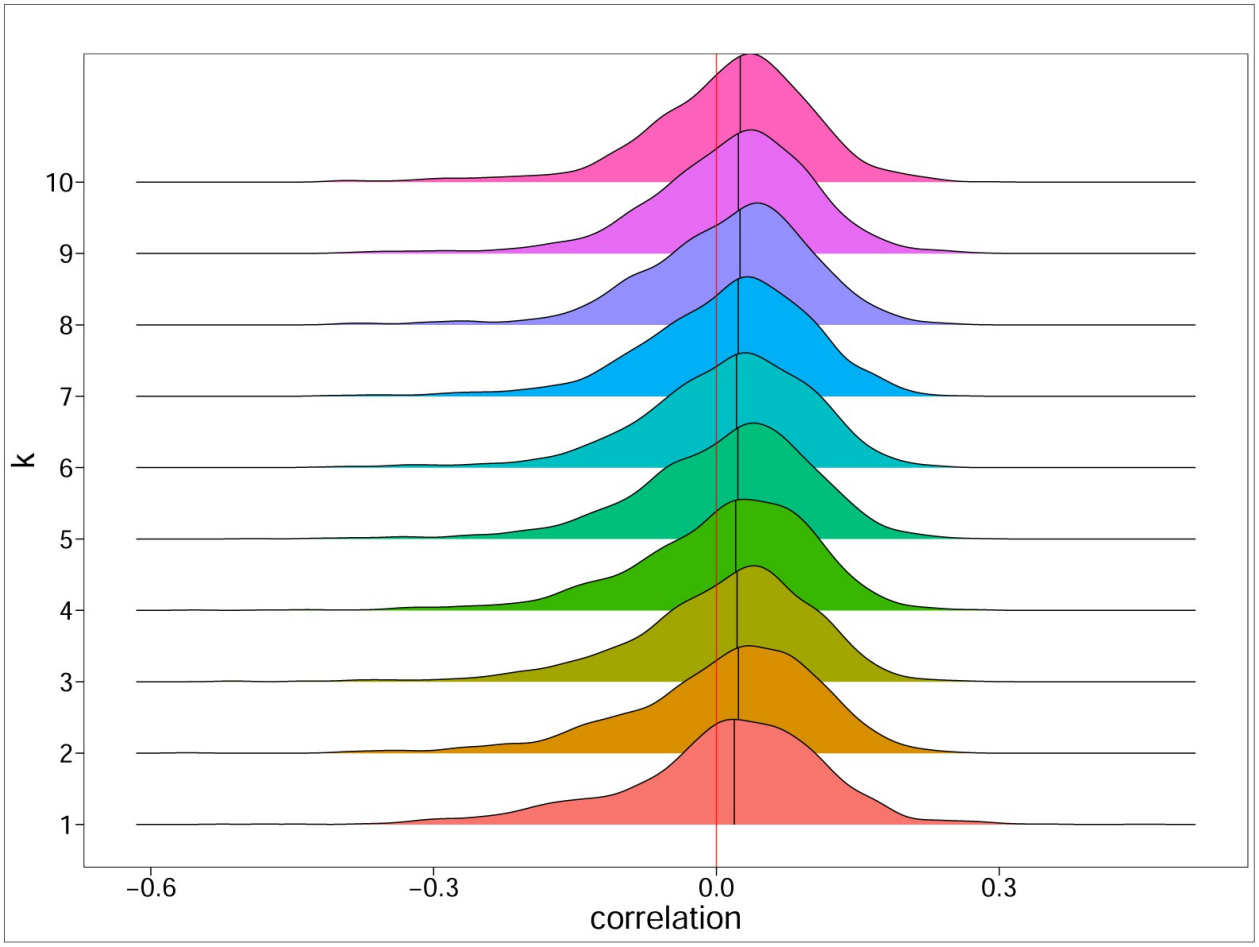
Density ridge plot of $Corr(Q_{j,t}^{\left(s\right)},|Y_{j,t+k}|)$, where *k* = 1, ⋯, 10; the solid black line represents the median of each distribution.

With the daily closing price *P*_*j*,*t*_ of stock *j* on trading day *t*, we defined the return *k* periods ahead as *Y*_*j*,*t*+*k*_ = *log*(*P*_*j*,*t*+*k*_) − *log*(*P*_*j*,*t*_) as described in Eq (11). Then, we correlated $Q_{j,t}^{\left(s\right)}$ with the absolute *k*-period return, |*Y*_*j*,*t*+*k*_|, where *k* = 1, …, 10 to investigate the predictability of the volatility and future losses using the local assortativity feature. We were interested here in the volatility of the stocks, and we correlated $Q_{j,t}^{\left(s\right)}$ with |*Y*_*j*,*t*+*k*_| because in statistical terms, volatility refers to the standard deviation of financial returns. Unfortunately, the standard deviation of a random variable (in our case the financial returns) is not observable. Therefore, to quantify the market fluctuation, we used the absolute returns here which can be regarded as proxies of the fluctuations [40]. Correlating $Q_{j,t}^{\left(s\right)}$ with |*Y*_*j*,*t*+*k*_| for stock *j* would provide evidence of how the local feature is related to volatility of a stock [41]. It can be observed in Fig 5 that in our case $Q_{j,t}^{\left(s\right)}$ and |*Y*_*j*,*t*+*k*_| were not linearly correlated. Regardless of the lag *k* under observation, $Q_{j,t}^{\left(s\right)}$ does not seem to provide significant predictive power for future absolute returns and thus for volatility.

#### $Q_{j,t}^{\left(s\right)}$ and future losses

Instead of correlating $Q_{j,t}^{\left(s\right)}$ with the absolute return |*Y*_*j*,*t*+*k*_|, we next focused only on trading days *t* + 1 when a loss occurred. In other words, we explored the multiple correlations between the local coefficient $Q_{j,t-l}^{\left(s\right)}$ for *l* = {0, ⋯, 5} on trading day *t* − *l*, and the loss on trading day *t* + 1, denoted as *L*_*j*,*t*+1_ in Eq (13). In this investigation, “*l* + 1” represents the number of lagged variables in the vector ($Q_{j,t-l}^{\left(s\right)},\ldots ,Q_{j,t}^{\left(s\right)}$) to correlate with *L*_*j*,*t*+1_. For example, if *l* = 2, we study the multiple correlation of the previous three standardized local assortativity ($Q_{j,t-2}^{\left(s\right)}$, $Q_{j,t-1}^{\left(s\right)}$, $Q_{j,t}^{\left(s\right)}$) to correlate with *L*_*j*,*t*+1_. The network degree of node *v*_*j*_ at time *t* − *l*, *d*_*j*,*t*−*l*_ was added alongside $Q_{j,t-l}^{\left(s\right)}$ to investigate whether the network statistics could complement each other and provide additional predictive power to future losses.

**Fig 6:**
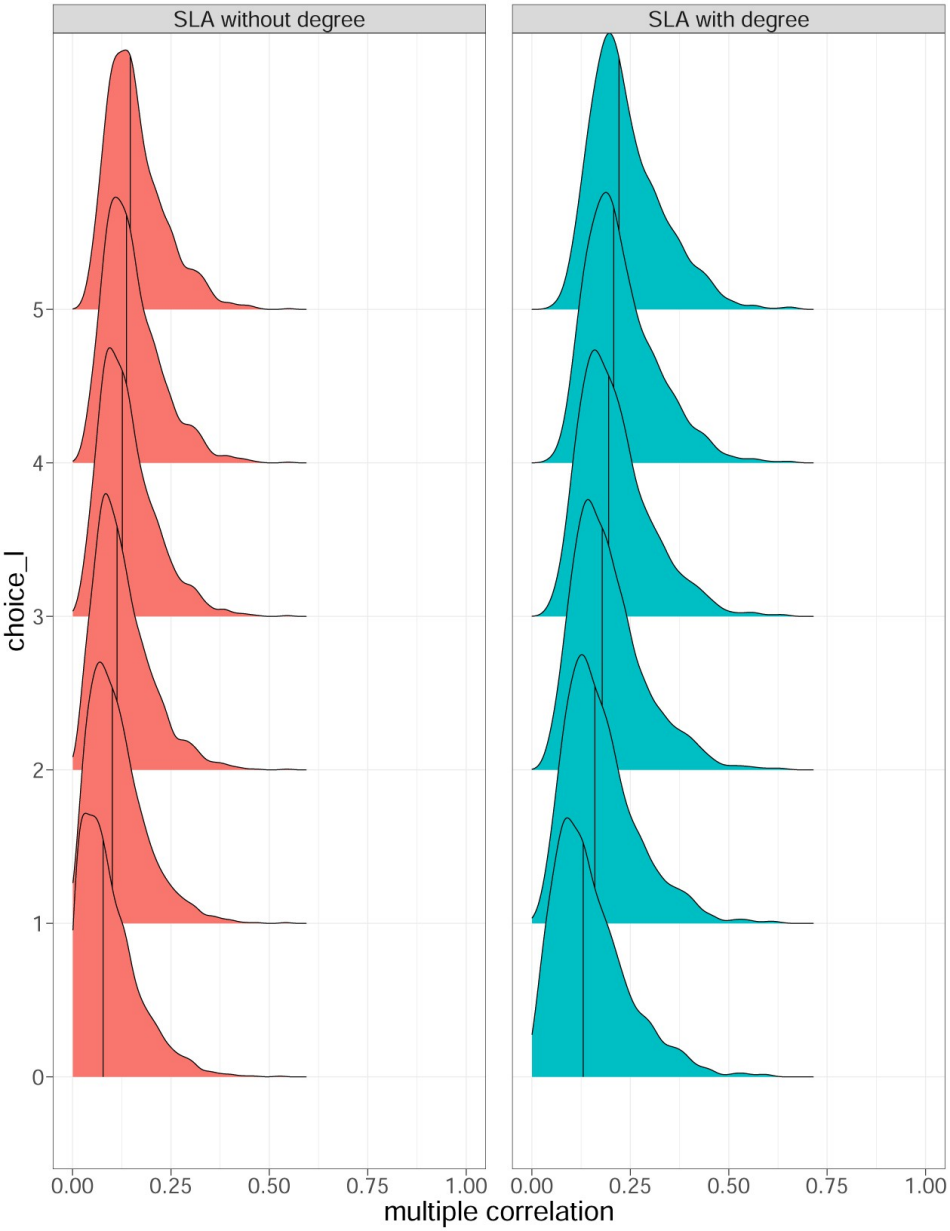
Density ridge plot of the conditional multiple correlation of $Q_{j,t-l}^{\left(s\right)}$, *l* = 0, ⋯, *N*, and the loss *L*_*j*,*t*+1_ (left panel), and the conditional multiple correlation of $Q_{j,t-l}^{\left(s\right)}$, *d*_*j*,*t*−*l*_, *l* = 0, ⋯, *N*, and the loss *L*_*j*,*t*+1_ (right panel), where *N* = 0, ⋯, 5; the solid black line represents the median of each distribution.

The density ridge plot in Fig 6 suggests a moderately positive correlation between $Q_{j,t-l}^{\left(s\right)}$ and *L*_*j*,*t*+1_. A stronger correlation with *L*_*j*,*t*+1_ emerged when more lags were included, in contrast to the previous case when we correlated $Q_{j,t}^{\left(s\right)}$ with |*Y*_*j*,*t*+*k*_| in Fig 5. The standardized local assortativity seemed to be more related to actual financial losses than to market volatility.

When *d*_*j*,*t*−*l*_ was added into the equation, the strength in correlation increased further. For example, the medians of the multiple correlations were 0.130 and 0.078 for *l* = 0, and 0.221 and 0.147 for *l* = 5, with and without *d*_*j*,*t*−*l*_, respectively. Combining the degree of nodes and the standardized local assortativity can give a median multiple correlation as high as 0.265, thus indicating mild predictability of the network statistics on financial losses.

#### Conditioned $Q_{j,t}^{\left(s\right)}$ and future losses

The correlation between $Q_{j,t}^{\left(s\right)}$ and *L*_*j*,*t*+1_ has suggested that the standardized local assortativity of financial networks tends to correlate with future loss. In Fig 4, the top panel gives the proportion of stocks with $Q_{j,t}^{\left(s\right)}>0.6$ on each day. When this proportion of locally assortative stocks with $Q_{j,t}^{\left(s\right)}>0.6$ is high, the financial market tends to adjust downward. Taking this one step further, we calculated the multiple correlations of $Q_{j,t}^{\left(s\right)}$ and *L*_*j*,*t*+1_ on days *t*, conditioned on $Q_{j,t}^{\left(s\right)}$ being greater than a threshold of 0.6. The main purpose was to pick out days when stock *j* exhibited a highly assortative pattern locally. When this occurred, we wanted to know whether the standardized local assortativity would help in detecting future losses more accurately.

**Fig 7:**
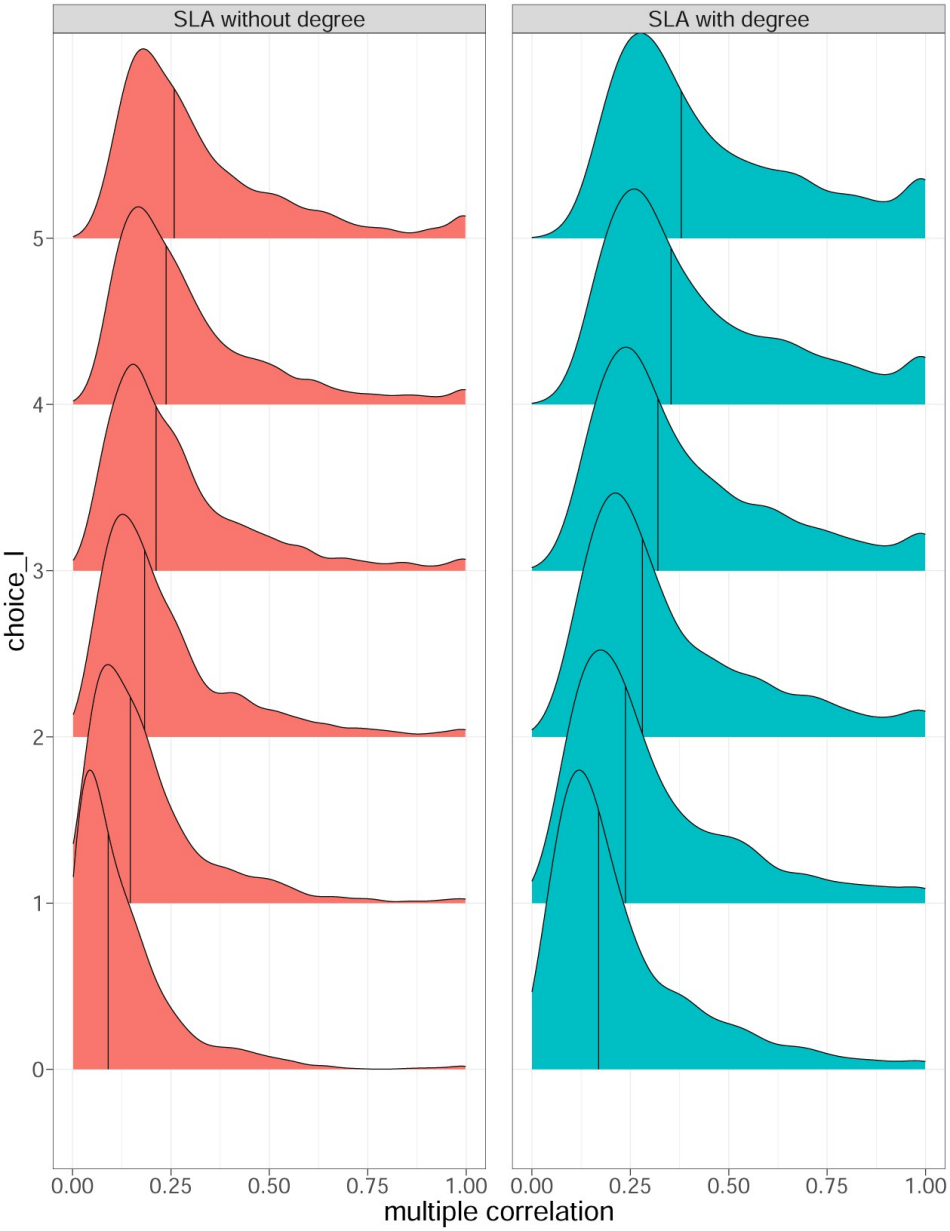
Density ridge plot of the conditional multiple correlation of $Q_{j,t-l}^{\left(s\right)}$, *l* = 0, ⋯, *N*, and the loss *L*_*j*,*t*+1_ given $Q_{j,t}^{\left(s\right)}>0.6$ (left panel), and conditional multiple correlation of $Q_{j,t-l}^{\left(s\right)}$, *d*_*j*,*t*−*l*_, *l* = 0, ⋯, *N*, and the loss *L*_*j*,*t*+1_ given $Q_{j,t}^{\left(s\right)}>0.6$ (right panel), where *N* = 0, ⋯, 5; the solid black line represents the median of each distribution.

Fig 7 displays a view similar to Fig 6, except the correlation was conditioned to when $Q_{j,t}^{\left(s\right)}$ was greater than 0.6. The results show a drastic increase in the multiple correlations between future loss and the respective lagged $Q_{j,t-l}^{\left(s\right)}$. As with the previous graph, we observe that the more lags we included, the stronger the positive correlation was. However, we also see that the distributions were heavily skewed when compared with those in Fig 6. On the left panel, when only $Q_{j,t-l}^{\left(s\right)}$ with five lags were included, the median of multiple correlations had increased to approximately 0.32. On the other hand, when *d*_*j*,*t*−*l*_ was added to the picture, in the right panel the median of multiple correlations was already close to 0.4 when five lags were included, suggesting that $Q_{j,t-l}^{\left(s\right)}$ and *d*_*j*,*t*−*l*_ for *l* = {0, ⋯, 5} may help predict future losses when the node at time *t* exhibits assortative behaviors. From the risk management perspective, the inclusion of a large number of lags is generally not recommended; however, the experiments show the potential predictive power for future losses by using the standardized local assortativity coefficient in dynamic financial networks.

## Discussion

With increasingly complex networks and the presence of clusters with their own degree of assortativity, we see the inadequacy of using a single global measure to quantify the assortative mixing of networks. We have shown a few networks with social and financial data in this research, all of which have indicated that local patterns are smoothed out if we simply use a global assortativity coefficient to summarize the networks.

A few research studies have suggested the use of local assortativity [34, 35]. However, there has not been a universal definition of how local assortativity should be calculated. Furthermore, the downside of the definition proposed by [35] faces the issue of unbounded upper ranges as the network size grows. To handle that shortcoming, we propose a standardized version of the previously defined local assortativity, with the coefficient lying within the range of -1 and 1, thus providing a neater summary representation of networks.

In this paper we tested the standardized local assortativity coefficient on several networks, from a generated community detection benchmark, to a real-world Facebook ego network, and to dynamic financial networks. In our LFR benchmark network, the global coefficient suggested neutral assortative mixing despite the presence of communities in the network. Facebook’s social network also showed that the global coefficient failed to capture the assortative nature of an ego network. The application of a standardized local measure, as is shown in Fig 2d, displays a more granular distribution for these networks. The comparison in Table 3 demonstrates a substantial discrepancy between the median $Q_{j,t}^{\left(s\right)}$ and the global assortativity in March 2020, signifying incredibly high systemic risk when the COVID-19 pandemic began to have a great impact on the world economy and financial markets. As is more evidence in Fig 4, the proportion of stocks having high values of $Q_{j,t}^{\left(s\right)}$ greater than 0.6 in our case might provide good early warning signals on severe market downturns.

Standardized local assortativity was also seen to provide predictive power in the chosen financial networks. Whereas the global assortativity at time *t* was unable to predict the future absolute returns, a proxy as future volatility, $Q_{j,t-l}^{\left(s\right)}$ did provide a higher predictive power in the case of a loss situation in the future. The standardized local assortativity coefficient $Q_{j,t-l}^{\left(s\right)}$, when in combination with other network statistics (i.e., network degree in our case), showed even higher predictive power for the future performance in terms of loss. The correlation became stronger when more lags of $Q_{j,t-l}^{\left(s\right)}$ were included. To further test out scenarios when networks were showing highly assortative patterns, we selected networks with $Q_{j,t}^{\left(s\right)}$ above the threshold of 0.6, and the predictability became even more obvious. When we included only the one lag of $Q_{j,t-l}^{\left(s\right)}$, the median correlation was already as strong as when we included five lags where no conditioning of $Q_{j,t}^{\left(s\right)}$ was applied, and the correlation continued to increase up to a median value of approximately 0.3 when five lags were included. The network degree provided an extra boost to the correlation when it was included: the median of multiple correlations went from approximately 0.25 if only one lag was included, to nearly 0.5 with five lags of $Q_{j,t-l}^{\left(s\right)}$. The results thus suggest the possibility of gaining extra insights from micro-features for financial risk management, if we construct networks out of the financial stock prices and observe the dates when the networks are highly assortative *locally*, we potentially will be able to foresee future losses and take preparedness action for risk management.

We saw that the standardized local assortativity coefficient complements other network statistics: when it is included, its correlation becomes stronger with future losses in dynamic financial networks. Whereas other network statistics have their own significance, we advocate the use of a local assortativity coefficient because it provides a summary of both the homophilic and resilient characteristics of a network.

We also may be able to draw implications for financial risks from the local assortativity coefficient. When nodes are more *locally* assortative, it implies that stocks will have a higher coherence of degrees with their neighbor nodes in the network, and we could use that coherence to provide early warning signals of severe market downturns or to predict a loss situation in the foreseeable future, as is shown in Figs 4 and 7. The visualization in Fig 3a shows that this high coherence of degrees or local assortative mixing can occur locally in many clusters that global assortativity cannot capture. The big mismatch of global assortativity and standardized local assortativity could be a potential indicator to inform us of unusual homophilic characteristics forming in a network in the future, and providing an early signal of increased systemic risk in financial markets. Further research needs to be conducted next on the modeling of local assortative patterns on financial risks.

## Conclusions

**Fig 8:**
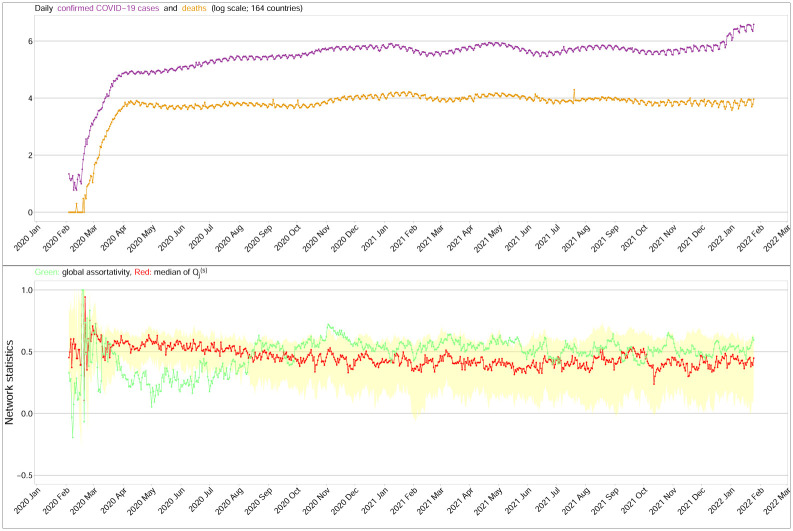
Top panel: time series plot of the number of confirmed cases of COVID-19 and deaths due to COVID-19 (in log scale) from 2020 to 2021. Bottom panel: global assortativity (green line) and the median $Q_{j,t}^{\left(s\right)}$ (red line), with its interquartile ranges highlighted by yellow regions of the dynamic pandemic networks in [30].

This study applied the standardized local assortativity coefficient on generated benchmarks in social networks, and in dynamic financial networks, but we believe it is possible that this concept can be extended to other networks in other fields, such as biology, epidemiology, and technology, to take into account the local features that otherwise can be easily omitted in complex networks. The results from the financial networks have suggested the importance of micro-features in risk management, and we believe that the same characteristics can be also found in other fields where groups of local nodes might help us answer important questions. Fig 8 shows the number of confirmed COVID-19 cases, deaths due to COVID, the global assortativity and the local assortative coefficient $Q_{j,t}^{\left(s\right)}$ obtained from the dynamic pandemic networks in [30]. When the COVID-19 pandemic was evolving quickly from March to July 2020, most of the values of $Q_{j,t}^{\left(s\right)}$ were larger than the corresponding global assortativity was. After July 2020, the trend of the median $Q_{j,t}^{\left(s\right)}$ and the trend of the global assortativity were similar. By following the trends of the standardized local assortativity of the dynamic pandemic networks especially when the local and global assortativities are very different, we may find hints as to when the COVID-19 pandemic is likely to be over.

## References

[1] Strogatz SH. Exploring complex networks. Nature. 2001;410(6825):268–276. doi: 10.1038/35065725 11258382

[2] Newman ME, Strogatz SH, Watts DJ. Random graphs with arbitrary degree distributions and their applications. Physical Review E. 2001;64(2):026118. doi: 10.1103/PhysRevE.64.026118 11497662

[3] Newman ME. Assortative mixing in networks. Physical Review letters. 2002;89(20):208701. doi: 10.1103/PhysRevLett.89.208701 12443515

[4] Newman MEJ. Mixing patterns in networks. Physical Review E. 2003;67(2). doi: 10.1103/PhysRevE.67.026126 12636767

[5] Newman ME. Coauthorship networks and patterns of scientific collaboration. Proceedings of the National Academy of Sciences. 2004;101(suppl 1):5200–5205. doi: 10.1073/pnas.0307545100 14745042 PMC387296

[6] Borgatti SP, Mehra A, Brass DJ, Labianca G. Network analysis in the social sciences. Science. 2009;323(5916):892–895. doi: 10.1126/science.1165821 19213908

[7] Bansal S, Read J, Pourbohloul B, Meyers LA. The dynamic nature of contact networks in infectious disease epidemiology. Journal of Biological Dynamics. 2010;4(5):478–489. doi: 10.1080/17513758.2010.503376 22877143

[8] Lin HH, Zhang LL, Yan R, Lu JJ, Hu Y. Network analysis of drug–target interactions: A study on FDA-approved new molecular entities between 2000 to 2015. Scientific Reports. 2017;7(1):12230. doi: 10.1038/s41598-017-12061-8 28947756 PMC5612934

[9] Li J, Zhou D, Qiu W, Shi Y, Yang JJ, Chen S, et al. Application of weighted gene co-expression network analysis for data from paired design. Scientific Reports. 2018;8(1):622. doi: 10.1038/s41598-017-18705-z 29330528 PMC5766625

[10] Chu AMY, Tiwari A, So MKP. Detecting early signals of COVID-19 global pandemic from network density. Journal of Travel Medicine. 2020;27(5):taaa084. doi: 10.1093/jtm/taaa084 32463088 PMC7542672

[11] Chu AMY, Chan JNL, Tsang JTY, Tiwari A, So MKP. Analyzing cross-country pandemic connectedness during COVID-19 using a spatial-temporal database: Network analysis. JMIR Public Health and Surveillance. 2021;7(3):e27317. doi: 10.2196/27317 33711799 PMC8088858

[12] Tiwari A, So MKP, Chong ACY, Chan JNL, Chu AMY. Pandemic risk of COVID-19 outbreak in the United States: An analysis of network connectedness with air travel data. International Journal of Infectious Diseases. 2021;103:97–101. doi: 10.1016/j.ijid.2020.11.143 33212255 PMC7668219

[13] Chu AMY, Chan TWC, So MKP, Wong WK. Dynamic network analysis of COVID-19 with a latent pandemic space model. International Journal of Environmental Research and Public Health. 2021;18(6). doi: 10.3390/ijerph18063195 33808764 PMC8003574

[14] Ng KC, So MKP, Tam KY. A latent space modeling approach to interfirm relationship analysis. ACM Transactions of Management Information Systems. 2021;12(2). doi: 10.1145/3424240

[15] Elliott M, Golub B, Jackson MO. Financial networks and contagion. American Economic Review. 2014;104(10):3115–3153. doi: 10.1257/aer.104.10.3115

[16] Song J, Zhang Z, So MKP. On the predictive power of network statistics for financial risk indicators. Journal of International Financial Markets, Institutions and Money. 2021;75:101420. doi: 10.1016/j.intfin.2021.101420

[17] So MKP, Chu AMY, Chan TWC. Impacts of the COVID-19 pandemic on financial market connectedness. Finance Research Letters. 2021;38:101864. doi: 10.1016/j.frl.2020.101864

[18] Chu AMY, Chan LSH, So MKP. Stochastic actor-oriented modelling of the impact of COVID-19 on financial network evolution. Stat. 2021;10. doi: 10.1002/sta4.408 34900251 PMC8646286

[19] So MKP, Chan LSH, Chu AMY. Financial network connectedness and systemic risk during the COVID-19 pandemic. Asia-Pacific Financial Markets. 2021;28(4):649–665. doi: 10.1007/s10690-021-09340-w

[20] Radicchi F, Castellano C, Cecconi F, Loreto V, Parisi D. Defining and identifying communities in networks. Proceedings of the National Academy of Sciences. 2004;101(9):2658–2663. doi: 10.1073/pnas.0400054101 14981240 PMC365677

[21] McPherson M, Smith-Lovin L, Cook JM. Birds of a feather: Homophily in social networks. Annual Review of Sociology. 2001;27(1):415–444. doi: 10.1146/annurev.soc.27.1.415

[22] Rand DG, Arbesman S, Christakis NA. Dynamic social networks promote cooperation in experiments with humans. Proceedings of the National Academy of Sciences. 2011;108(48):19193–19198. doi: 10.1073/pnas.1108243108 22084103 PMC3228461

[23] Lusseau D, Newman MEJ. Identifying the role that animals play in their social networks. Proceedings of the Royal Society of London Series B: Biological Sciences. 2004;271(suppl_6):S477–S481. doi: 10.1098/rsbl.2004.0225 15801609 PMC1810112

[24] Fu F, Nowak MA, Christakis NA, Fowler JH. The evolution of homophily. Scientific Reports. 2012;2(1):845. doi: 10.1038/srep00845 23150792 PMC3496167

[25] Bergstrom TC. The algebra of assortative encounters and the evolution of cooperation. International Game Theory Review. 2003;05(03):211–228. doi: 10.1142/S0219198903001021

[26] Sterbenz JPG, Cetinkaya EK, Hameed MA, Jabbar A, Rohrer JP. Modelling and analysis of network resilience. In: 2011 Third International Conference on Communication Systems and Networks (COMSNETS 2011); 2011. p. 1–10.

[27] Smith P, Hutchison D, Sterbenz JPG, Scholler M, Fessi A, Karaliopoulos M, et al. Network resilience: A systematic approach. IEEE Communications Magazine. 2011;49(7):88–97. doi: 10.1109/MCOM.2011.5936160

[28] Barclay VC, Smieszek T, He J, Cao G, Rainey JJ, Gao H, et al. Positive network assortativity of influenza vaccination at a high school: Implications for outbreak risk and herd immunity. PLOS ONE. 2014;9(2):e87042. doi: 10.1371/journal.pone.0087042 24505274 PMC3914803

[29] So MKP, Tiwari A, Chu AMY, Tsang JTY, Chan JNL. Visualizing COVID-19 pandemic risk through network connectedness. International Journal of Infectious Diseases. 2020;96:558–561. doi: 10.1016/j.ijid.2020.05.011 32437929 PMC7207126

[30] So MKP, Chu AMY, Tiwari A, Chan JNL. On topological properties of COVID-19: Predicting and assessing pandemic risk with network statistics. Scientific Reports. 2021;11:5112. doi: 10.1038/s41598-021-84094-z 33664280 PMC7933279

[31] Iyer S, Killingback T. Evolutionary dynamics of the traveler’s dilemma and minimum-effort coordination games on complex networks. Phys Rev E. 2014;90:042134. doi: 10.1103/PhysRevE.90.042134 25375465

[32] Iyer S, Killingback T. Evolution of cooperation in social dilemmas on complex networks. PLOS Computational Biology. 2016;12(2):1–25. doi: 10.1371/journal.pcbi.1004779 26928428 PMC4771135

[33] Iyer S, Killingback T, Sundaram B, Wang Z. Attack robustness and centrality of complex networks. PLOS ONE. 2013;8(4):1–17. doi: 10.1371/journal.pone.0059613 23565156 PMC3615130

[34] Peel L, Delvenne JC, Lambiotte R. Multiscale mixing patterns in networks. Proceedings of the National Academy of Sciences. 2018;115(16):4057–4062. doi: 10.1073/pnas.1713019115 29610344 PMC5910813

[35] Thedchanamoorthy G, Piraveenan M, Kasthuriratna D, Senanayake U. Node assortativity in complex networks: An alternative approach. Procedia Computer Science. 2014;29:2449–2461. doi: 10.1016/j.procs.2014.05.229

[36] Leskovec J, Krevl A. SNAP datasets: Stanford large network dataset collection; 2014. http://snap.stanford.edu/data.

[37] Clemente GP, Grassi R, Hitaj A. Asset allocation: New evidence through network approaches. Annals of Operations Research. 2021;299:61–80. doi: 10.1007/s10479-019-03136-y

[38] Lancichinetti A, Fortunato S, Radicchi F. Benchmark graphs for testing community detection algorithms. Physical Review E. 2008;78(4). doi: 10.1103/PhysRevE.78.046110 18999496

[39] Leskovec J, Mcauley J. Learning to discover social circles in ego networks. In: Pereira F, Burges CJC, Bottou L, Weinberger KQ, editors. Advances in Neural Information Processing Systems. vol. 25. Curran Associates, Inc.; 2012.

[40] So MKP, Chu AMY, Lo CCY, Ip CY. Volatility and dynamic dependence modeling: Review, applications, and financial risk management. Wiley Interdisciplinary Reviews: Computational Statistics; p. e1567.

[41] So MKP, Xu R. Forecasting intraday volatility and Value-at-Risk with high-frequency data. Asia-Pacific Financial Markets. 2013;20(1):83–111. doi: 10.1007/s10690-012-9160-1

